# 加速康复外科评价指标：病人报告结局在胸外科的临床应用现状与进展

**DOI:** 10.3779/j.issn.1009-3419.2019.03.08

**Published:** 2019-03-20

**Authors:** 诚 沈, 珏 李, 鹏飞 李, 国卫 车

**Affiliations:** 610041 成都，四川大学华西医院胸外科 Department of Thoracic Surgery, West China Hospital, Sichuan University, Chengdu 610041, China

**Keywords:** 加速康复外科, 病人报告结局, 健康相关生活质量, 胸外科, Enhanced recovery after surgery, Patient reported outcomes, Health related quality of life, Thoracic surgery

## Abstract

加速康复外科临床应用的良好效果体现在降低围手术期并发症的发生率并缩短住院时间，但对围术期的患者症状管理及术后患者生活质量的关注不够高。从患者报告的资料角度评估临床疗效越来越受到重视。结合目前国内外关于病人报告结局的研究成果，本文系统论述了病人报告结局的概念内涵、研究意义及在胸外科的临床应用现状，提出借鉴国外病人报告的临床结局研究模式，开展有中国特色的结合学科特点的相关研究，并对已有的文献报告进行总结及分析。

## Abstract (en)

The good effect of enhanced recovery after surgery is reflected in reducing the incidence of perioperative complications and shortening the hospital stays. However, the concern for the management of perioperative patients and the quality of life of patients after surgery is not high enough. Evaluating clinical efficacy from the perspective of patient-reported data has received increasing attention. Combining the current domestic and foreign research results on the outcome of patient reports, this article systematically discusses the concept connotation, research significance, and clinical application of thoracic surgery for the outcome of patient reports with proposing a clinical outcome research model that draws on foreign patient reports to develop Chinese characteristics. Combine the related research of discipline characteristics, and summarize and analyze the existing literature reports.

随着医学技术的发展及医疗模式不断改进，患者对生存质量的要求也越来越高。同时，这也不断提醒着临床医生，患者的主观感受与临床客观性指标一样对评估临床疗效起着重要作用。因此从患者的临床症状、心理状态、社会生活、自身状态满意度及临床疗效满意度等多维度多层次评价疾病的临床疗效越来越受重视，而病人报告结局（patient reported outcomes, PROs）作为一种通过捕捉与病人健康相关的某些概念的评估方法，可以为临床的治疗效果提供了一种评价的手段，其具体是指可通过访谈、自评问卷或其他数据捕捉工具，包括有关病人日常生活、健康状态和治疗措施等方面的日志，得到直接来自于病人报告的相关资料^[1, 2]^。

此外，伴随着对围术期患者病理生理认识的提高及微创外科、疼痛控制等技术水平的提高，外科治疗理念也有所改变。加速康复外科（enhanced recovery after surgery, ERAS）是医学理论和外科技术发展的必然结果。其理念是指采用循证医学证据的围手术期优化组合措施，以最大程度地减轻手术相关应激，加速患者术后康复进程，降低并发症的发生率，改善外科患者预后，从而提供更优质的医疗服务^[3]^。优化围手术期患者的临床情况能够减少由于手术引起的身体和精神方面的应激，以促进患者更好的康复^[4]^。2015年以后出现的快速康复外科应该用患者症状恢复作为目的；有研究者认为，ERAS的效果评定多是从医生角度进行评价，不能准确地反映患者机体状况和感受，而提出PROs作为评定是否快速康复的指标，但目前此类研究在胸外科结合ERAS理念的临床应用中尚少^[5]^。本文就PROs的发展及其在胸外科临床实践中结合ERAS理念应用的最新进展作一综述。

## 1 PROs的概况

### 1.1 PROs的概念

PROs是从病人的角度获取临床资料，是病人健康状态的具体反映，在临床评价体系中具有重要作用^[5]^。其重点是以心理学理论和方法为基础，以可靠、正确以及能得到同行认同的经验为支撑，是评估临床结局变化的科学手段，可以用于评估患者的主观感受、自觉症状以及与治疗措施的选择密切相关的患者满意度^[6-8]^。综上，病人报告结局是在病人就诊及其治疗期间，临床医生询问患者的主观感受、症状以及客观事件的变化情况，依此协助诊断疾病、了解病情和判断治疗效果，所有此类由患者自身报告的与健康相关的结局的各种信息即是PROs^[9]^。

### 1.2 PROs的内涵

随着人们对PROs应用于疗效评价的不断认识，人们对PROs的内涵研究也越来越深入。分歧协调委员会（The Harmonization Coordination Committee Representing）在2001年组织召开的“基于病人报告结局研究的重要问题”（在http://www.eriqa-project.com/pro-harmo/home.html网页上可检索到相关资料）上总结到以下四个方面。

#### 1.2.1 疾病活动的独特迹象

绝大多数非器质性疾病或是功能性疾病如神经官能症、抑郁症、更年期综合征和失眠等实验室检查指标可能出现阴性结果，而此时患者提供的自觉症状是诊断这些疾病的唯一证据。实际上，患者的主观感受和实验室指标在某些疾病的诊断方面是互为补充。

#### 1.2.2 从本质上评估疗效

病人的自我感受是主要或唯一的疾病活动标志，如果用于临床试验的药物对患者的自觉症状有所改善，并保证治疗安全，那么患者的感受即PROs可以作为评估疗效终点的本质。

#### 1.2.3 用于最佳治疗方案的评价与选择

PROs可以提供治疗疾病的额外信息，除了反映患者的综合健康状况以外，更重要的是可用于药物疗效和治疗方案的评价与选择。

#### 1.2.4 解释临床结果

PROs以病人的利益为出发点，可以解释生理指标的变化对患者健康状况的影响。在肿瘤相关的呼吸系统、心血管系统以及胃肠道疾病，有关组织已经用PROs来指导临床治疗，并使其成为治疗决策的主要因素之一。

### 1.3 PROs的研究意义

#### 1.3.1 强调“以病人为中心”的理念

“以病人为中心”的理念，突出了在评估医疗服务中要从病人的角度来看问题，若缺乏了病人对接受治疗的满意度、主观症状及生活质量等情况的了解，则会使医疗评估缺乏合理性和完整性^[10]^。同时，以病人为中心还意味着病人自己要对是否继续接受治疗作出决定和自我管理。临床医生通过PROs让病人根据功效、副作用和耐受性选择治疗手段^[11, 12]^。

这样的理念与ERAS的观念不谋而合。通过对个体化患者围手术期进行风险评估和干预，优化治疗共存病症包括心脑血管、呼吸和泌尿等系统疾病，了解和提前处理患者存在的社会和行为因素，比如对烟草和酒精的依赖等，减少术中和术后患者身体对外科手术的严重应激反应，达到降低术后并发症和病死率，缩短患者住院时间和住院费用，真正从病人角度出发^[13, 14]^。

#### 1.3.2 更为全面地获得诊疗信息

医生所获得的临床结果往往得到的是一些客观的指标，而较少获得病人主观陈述的心理感受或情绪状态。术后生命期的延长，使得患者对生活质量的要求变得更高，但是手术相关症状管理却常常被忽视，尤其是患者出院后发生的相关症状无法得到很好的解决。手术相关症状管理是ERAS的重要组成部分，客观评价肺癌患者手术相关症状，对于了解其产生的原因（麻醉引起、手术引起或并发症引起）以及正确治疗都有重要临床意义^[15]^。而通过PROs可以得到病人完整的资料，包括生理和心理方面。尤其是对于术后随访病人的重视，采用客观评估和有效管理（治疗）的方法，满足了病人不单纯以临床结果为基础，而是自身状态综合性和全面性的反映，由此可以获得更全面的诊疗信息以指导临床。

### 1.4 PROs的研究现状

近年来，随着医学模式的改变，由国际药物经济与结果研究协会、欧洲生存质量评估协调处、美国食品及药物管理局与健康相关生存质量工作组和国际生存质量研究协会共同组成的统筹委员会提出，临床疗效评价应包括4个方面的内容：临床医务人员报告资料、生理报告资料、照顾者报告资料和患者报告资料^[16]^。随着医学模式的转变，在临床疗效评估方面，除以往医学研究中一直重视的临床医务人员报告资料和生物学报告资料外，人们越来越重视病人报告资料在疾病诊疗及疗效评价过程中的作用。在我国，关于PROs的研究还处于初级阶段。目前国内的PROs的研究项目主要集中于中医临床疗效评价中，具体包括在慢性阻塞性肺疾病、类风湿关节炎、女性压力性尿失禁病人等的研究^[17-20]^。值得注意的是，目前有关PROs的大部分量表都是从国外直接引进并在一定程度上进行了修订，然而鉴于西方文化和我们的差异，部分量表的测试内容很难真实反映我们患者的具体情况。

## 2 PROs在胸外科临床中的应用

### 2.1 PROs在胸外科中应用的背景

在胸外科手术中，对于病情的评估主要集中在对生存率和发病率的客观测量上。人们普遍认为，尤其是在癌症治疗方面，最好的疗法是为了提供最长的生存时间。生存率、围手术期死亡率和并发症发生率的结果是客观的，比较容易衡量。然而，接受胸外科手术的患者会经历大量的术后症状，这些症状在这些分析中并没有被完全考虑到。这些症状有些是不明确的，比如疼痛、疲劳、情绪低落和焦虑。另一些是特定于疾病或器官的，例如呼吸困难、吞咽困难和胃肠痉挛。无论如何，这些症状都有一定程度的主观性。最终，提供“以病人为中心”的最佳护理方案将需要更多地关注到高质量的健康相关生活质量（health-related quality of life, HR-QOL）结果研究。最准确的评估和衡量这些症状的方法就是利用PROs直接从病人处收集这些数据。

在过去，以HR-QOL为研究对象的研究，其主观性常引起研究者对此类研究有效性的审视。其实这往往是由于方法上的问题，例如在PROs测量方面缺乏标准化，对症状的微妙变化以及在数据测量和耗时调查的管理上的困难。因此，大多数胸外科研究都集中在比较容易量化的客观指标上。毫无疑问，这些客观的结果研究是至关重要的，因为它们构成了治疗评估的基础，尤其是在肿瘤护理方面，它们主导了研究工作。然而，这种方法可能导致对治疗的不完整评估。仅靠生存率和并发症发生率并不能完整的评估术后患者的诊治全过程。在许多方面，HR-QOL往往更受患者的关注，也更与患者自身息息相关，而不是单纯在比较治疗方案时出现的5年生存率的微小差异。

### 2.2 PROs在胸外科临床应用中的具体实施

PROs在胸外科相关临床中也有所应用^[21-23]^。2013年Gazala等^[24]^做了一项有关胸腔镜手术（video-assisted thoracic surgery, VATS）和开胸手术后患者PROs的系统评价与荟萃分析。共纳入了5项有效研究，最终结果发现接受VATS手术的患者PROs评价结果好于开胸的患者，其PROs具体的评估量表采用的是HR-QOL量表。

2015年发表在*J Thorac Cardiovasc Surg*杂志的一篇研究^[5]^引起了大家的重视。该研究旨在通过使用纵向PROs评估，来定义肺癌患者行胸部手术的术后症状恢复情况，更好地指导ERAS理念在临床的应用。其纳入了60例分期为I期或者II期的非小细胞肺癌患者，收集了在术前到术后3个月的多种症状结果。手术方式包括了标准开胸手术和VATS手术。其使用的量表为MD安德森症状评估量表（MD Anderson Symptom Inventory, MDASI）。最终的评价结果发现，最为严重的5种症状分别是疲劳、疼痛、气短、失眠和困倦。对于接受VATS的患者（8 d）其疼痛恢复到正常状态的时间相较于开胸手术的患者（18 d）要更快一些（*P*=0.022）。术前PROs评估状态表现差的患者，其术后病人报告结局中发生疼痛的概率会更高（*P* < 0.05）。手术相关症状管理是ERAS的重要组成部分，尤其是通过PROs的评价方式对于患者术后疼痛症状的关注，不仅真实可靠地反映了患者自身的恢复状态，也更加有利于我们在围术期采取相关的措施进行提前的预防及精准治疗，同时也有利于我们优化ERAS理念在围术期症状管理中的流程，结合患者最为常见的术后症状进行及时的处理和预防，最终使患者更为受益，提高了生活质量^[5]^。

Berman等^[8]^提出他们将PROs评估体系与生存护理计划（survivorship care plans, SCPs）相结合。OncoLife和LIVESTRONG护理计划是基于互联网的一种新兴SCPs，作者通过纳入原发性肺癌的患者，基于病人报告结局的治疗方式和放化疗后的毒性反应数据均被记录。最终的数据收录了689份来自于患者自己的报告总结。其中，多数为居住在美国的白人（85.9%），接受化疗者占75.8%，放疗者为54.7%以及手术的患者占54.4%。神经认知症状（如疲劳、认知改变）是最常见的（48.8%），其次是肌肉骨骼/皮肤病症状（14.1%）和胸部症状（13.5%）。该研究也提示我们，为了获得更为全面的数据，基于网络的多中心患者PROs是可行的，然而在研究过程中依然存在不足，例如，患者在被要求做出回应时，仅仅只是回答“是”或“否”的答案，结果没有被要求量化，特别是有关生活质量方面的问题。因此，更好地量化问题答案将有益于阐明问题的严重程度。

Khullar等^[25]^也提出目前胸外科医师学会（The Society of Thoracic Surgeons, STS）的确是缺少病人报告结局的数据库。他们利用美国国立卫生研究院（National Institutes of Health, NIH）的病人报告结局测量信息系统（PatientReported Outcome Measurement Information System, PROMIS）^[26]^收集了127例患者术前1个月及肺癌术后6个月的PROs评估结果发现，最常见的手术方式是VATS（55%）。在第一次术后访视时发现，疼痛、疲劳和失眠显著增加以及身体机能下降。6个月随访结果提示患者已经基本恢复到了基线水平。类似的研究还包括Louie等^[27]^的团队比较了立体定向放疗（stereotactic body radiation therapy, SBRT）与手术切除治疗Ia期非小细胞肺癌的疗效。这项探索性分析发现，HR-QOL与间接成本在SBRT中更占优势。有研究^[28, 29]^在2017年提出了基于在线调查问卷系统（QTool），使用PROs评估VATS和SBRT如何影响HR-QOL，也希望这些结果能够同时帮助患者和临床医生在围术期管理方面做出正确的决策并改善相关问题。在肺移植方面，利用PROs对患者术后进行相关管理也愈来愈多。有研究^[6, 30]^提出，通过12身心健康成分得分简表、气道问卷20修订版、欧洲问卷5D以及老年抑郁量表对患者进行测评，结果提示使用ECMO的患者移植术后HR-QOL有明显的改善。

上述相关研究表明，PROs的评测优点可以体现在获得病人完整的资料，尤其是对于术后随访病人的重视，采用客观评估和有效管理（治疗）的方法，满足了病人不单纯以临床结果为基础，而是自身状态综合性和全面性的反映，由此可以获得更全面的诊疗信息以指导临床。然而，关于PROs在国内胸外科方面的研究还处于初级阶段。此外，现有的用于PROs的大部分量表都是从国外引进的修订量表，一些物理测量方法可以通用，但是有些关于日常生活和社会生活方面的问题，从语言思维、价值观到文化生活习俗都是存在差异的。因此，用西方的一些生存质量量表来测量我国病患的某些特性，很难测出他们真实的自我感受。

## 3 PROs联合ERAS在临床中的应用

ERAS是医学理论和外科技术发展的必然结果，它不但关注减少对机体的应激反应，同时也重视对手术进行风险评估和干预，优化治疗共存病症包括心血管、呼吸系统和/或肾脏疾病，同时治疗、维持患者在围手术期重要器官功能，了解和处理患者存在的社会和行为因素，进而达到临床上降低并发症和缩短住院时间的目的^[3, 4, 31]^。

同时，当前各个学科应用最多的是降低术后并发症和缩短住院时间，作为评价ERAS方案可行与否的标准。但也有研究者^[32, 33]^认为，ERAS的效果评定多是从医生角度进行评价，不能准确反映患者机体状况和感受，尤其是术后症状的管理，而提出PROs作为评定是否快速康复的指标。患者术后存在的种种症状严重影响了患者的医疗体验及就医满意度，也降低了治疗依从性。在实际的医疗工作中往往会出现患者由于伤口疼痛、咳嗽难止等原因拒绝出院，甚至导致医患冲突。减少手术相关症状对患者的困扰，需要医护人员对手术相关症状进行客观评估和有效管理。肺癌患者的症状一般可以分为生理症状和心理症状两部分，产生症状的原因则可能来自于三个部分。首先肿瘤对压迫周围组织或对血管的侵犯会带来一部分症状；其次目前的治疗手段，无论是手术治疗或者放疗与化疗，在控制疾病的同时又会带来新的症状；最后患者在确诊肺癌后的心理负担以及治疗过程中情绪的变化也会导致心理症状和部分生理症状^[21, 34]^。

目前，ERAS已在骨科、胃肠外科、乳腺外科、心胸外科、妇产科等多个外科领域开展且取得显著的临床结果。国内外已发布多种术后ERAS指南或专家共识。而PROs联合ERAS在外科临床的应用也逐渐成为热门研究的内容。在一项PROs在骨科ERAS的应用的系统评价中指出，共有2, 208例行髋关节和膝关节成形术的患者纳入讨论，结果发现与传统治疗相比，生活质量评分在运用ERAS后的1年内都逐渐提高^[35]^。对于行结直肠癌手术的患者，ERAS方案不仅可以减少术后并发症的发生，同时也缩短了患者住院天数。Shida等^[36]^研究者提出，早期出院的患者应当使用PROs的方法来评估患者接受ERAS方案后的反馈情况。94例在ERAS方案下行结直肠癌手术的患者接受了恢复质量评分（40-item quality of recovery score, QoR-40）的评估。以术前QoR-40的评分作为基线，结果发现手术患者在术后第1天和术后第3天的评分是低于平均值的（在基线水平以下），到了第6天，评分已经在恢复了。到了术后1个月，评分已完全达到甚至超过了基线水平。其中，年轻患者相比于老年患者在术后第1天的结果中评分要更低一些。在膀胱癌根治术研究中，利用MD安德森症状评估量表对383例运用了ERAS方案的患者进行了从术后第1天-第5天的PROs评价^[37]^。最终提示，相比于传统护理组，ERAS组在疼痛减轻、疲乏、口干和失眠方面均有较为明显的缓解，因此，这也充分说明了ERAS的意义以及PROs在对围术期治疗效果评价方面的成效。

## 4 问题与展望

随着对提供高质量“以患者为中心”的护理的关注越来越多，PROs越来越被许多社会和机构所认可和推荐。例如，当患者自身出现一些感觉上的细微变化，包括吞咽困难或呼吸困难等，术前完成相应PROs评估将可能是临床问题的早期预警信号。其次，通过术后常规进行PROs的方案，将有助于早期认识到问题的存在以便进行早期干预。此外，对PROs进行完整的多中心的前瞻性客观评估将为胸外科医生和患者提供术后HR-QOL针对症状改变的治疗方案。然而，PROs在国内的开展依然处于探索阶段，很多量表都是国外研究机构的讨论结果，是否一定适合于国内，还有待商榷。

总之，微创外科及其体系完善是ERAS发展的动力，医护一体和多学科协作是ERAS顺利实施的保障，在开展ERAS方案的同时，完善PROs在ERAS方案中的应用，借鉴国外PROs研究模式，开展有中国特色的PROs测量，建立计算机应用的健康评价系统，开发针对客观反映中国人群胸外科手术后相关症状评估量表，必将对ERAS方案在胸外科临床实际应用和实施的完善和发展起到积极地推进作用。
